# Ozone Treatment for the Management of Caries in Primary Dentition: A Systematic Review of Clinical Studies

**DOI:** 10.3390/dj12030069

**Published:** 2024-03-06

**Authors:** Federica Veneri, Tommaso Filippini, Ugo Consolo, Marco Vinceti, Luigi Generali

**Affiliations:** 1 Unit of Dentistry & Oral-Maxillo-Facial Surgery, Department of Surgery, Medicine, Dentistry and Morphological Sciences with Transplant Surgery, Oncology and Regenerative Medicine Relevance (CHIMOMO), University of Modena and Reggio Emilia, 41124 Modena, Italy; ugo.consolo@unimore.it (U.C.); luigi.generali@unimore.it (L.G.); 2 PhD Program in Clinical and Experimental Medicine, Department of Biomedical, Metabolic and Neural Sciences, University of Modena and Reggio Emilia, 41124 Modena, Italy; 3 Environmental, Genetic and Nutritional Epidemiology Research Center (CREAGEN), Section of Public Health, Department of Biomedical, Metabolic and Neural Sciences, University of Modena and Reggio Emilia, 41124 Modena, Italy; tommaso.filippini@unimore.it (T.F.); marco.vinceti@unimore.it (M.V.); 4 School of Public Health, University of California Berkeley, Berkeley, CA 94704, USA; 5 Department of Epidemiology, Boston University School of Public Health, Boston, MA 02118, USA

**Keywords:** children, deciduous teeth, dental caries, minimally invasive dentistry, ozone, pediatric dentistry

## Abstract

Dental caries in children is a frequent and debilitating condition, whose management is often challenging. The aim of this systematic review was to investigate the effectiveness of ozone applications for the treatment of caries in primary dentition. According to PRISMA guidelines, a systematic literature search was performed up to 6 January 2024. Clinical studies using ozone to treat caries of deciduous teeth were considered for inclusion. Out of the 215 records retrieved, seven studies were eventually included in the review, all of which used gaseous ozone. Four studies were judged at high risk of bias, two at low risk, and one of some concerns. The great heterogeneity of designs, outcomes, and protocols made it impossible to conduct a meta-analysis. Despite some limitations, the evidence yielded by the included studies suggests that ozone application, regardless of the protocol applied, is comparable to other interventions in terms of clinical outcomes and anti-bacterial activity, with no reported adverse effects and good patient acceptance. Therefore, ozone application may be a non-invasive approach to treat caries in primary dentition, especially in very young and poorly cooperative patients. Further standardized and rigorous studies are, however, needed to identify the best clinical protocols for this specific field.

## 1. Introduction

Dental caries is one of the most common chronic diseases in the pediatric population, affecting 60% to 90% of school children, and it is often related to low socioeconomic status [1]. Untreated caries in deciduous teeth is the 10th most prevalent condition, involving approximately 621 million children globally, indicating that the unmet treatment need is exceptionally high [2,3]. Untreated worsening dental caries can negatively impact a child’s oral and general health, as well as the quality of life both of the child and their family, frequently causing the impairment of sleeping and eating habits, growth patterns, school attendance, and socialization [4,5].

Treatment of caries affecting primary dentition can be particularly challenging, due to technical reasons, including specific anatomic and morphological features, which can influence the predictability of the treatments [6]. Furthermore, young patients may present with cooperation and compliance issues, resulting in sub-optimal clinical operative settings, where proper field isolation cannot be achieved or the operational timing needs to be limited, thus prompting the need for reliable, rapid, and effective treatments [7,8,9].

In recent years, non-invasive or minimally-invasive approaches have spread widely in many medical and dental fields, especially in pediatric dentistry, with the implementation of more biologic atraumatic techniques [10,11] Modern minimally invasive approaches include selective or stepwise carious removal, also using atraumatic manual techniques along with chemo-mechanical procedures, non-restorative treatments, such as fluoride varnish or silver diamine fluoride applications, and new suitable restorative solutions and materials with bioactive properties [12,13,14,15,16,17]. Overall, these approaches allow more conservative and less time-consuming procedures, requiring less need for anesthetics and causing reduced post-operative discomfort [6,18,19]. Among other minimally invasive strategies, ozone applications have been suggested for the management of carious lesions [20]. Ozone (O_3_) is a naturally occurring compound, consisting of three oxygen atoms deriving from the conversion of oxygen by ultraviolet radiation. Although not being a radical molecule, ozone is a powerful oxidizing agent that has broad-spectrum antimicrobial properties, as well as the ability to promote healing processes and to modulate inflammation at low medical concentrations, through the activation of protective antioxidant pathways, thus showing therapeutic effects in many diseases and medical fields [21,22,23,24,25,26,27].

Ozone applications in dentistry were introduced as early as the first half of the 1900s, but despite being abandoned for some time due to the risk of inhalation toxicity and the difficulty in obtaining optimal gas concentrations without dispersion, modern technologies and appropriate delivery and application techniques have allowed us to overcome such issues [28].

Ozone-induced oxidation is expected to counteract cariogenic biofilm, reducing bacterial count and consequently arresting or reversing the progression of dental caries, and therefore providing an alternative management strategy to the traditional “drill and fill” approach [20]. 

The anti-bacterial effect is explained by ozone-induced oxidation, which damages the cell wall and cytoplasmic membrane of microbial cells, increasing their permeability to ozone molecules and leading to cell lysis and death [20,29]. Moreover, ozone has shown promising properties for dentine and enamel remineralization by damaging the biomolecules of carious tissue and removing proteins from the demineralized surfaces, making them more pervious and ensuring greater perfusion of remineralizing agents and mineral ions [30]. 

For the aforementioned reasons, ozone has been proposed as a valid option for the treatment of dental caries. A meta-analysis conducted in 2020 on randomized clinical trials (RCTs) examined its use in both primary and permanent dentition and overall did not find sufficient evidence of efficacy to recommend the use of ozone, compared with other strategies [20]. The present review focused on the specific evaluation of ozone therapy in primary dentition and updated the relevant literature, adding relevant studies to the three RCTs examined in the previous review, also taking into account that a young age and lack of compliance may prevent the use of other strategies, thus limiting the possibility of conducting randomized controlled trials [30,31]. The rationale for this review was also the lack of invasiveness of this methodology, therefore suggesting the need to assess ozone therapy as a valid and suitable alternative in pediatric dentistry, especially when other conventional procedures may be limited or preferentially postponed for a variety of reasons [30,31]. Concerning this, the specific clinical context (e.g., age, compliance level, characteristics of the primary teeth and clinical evolution of caries) also has to be taken into account when considering the use of ozone for the treatment of dental caries. Therefore, this study aimed at assessing the overall effectiveness of ozone applications for the management of caries of primary dentition, according to the available clinical evidence. 

## 2. Materials and Methods

### 2.1. Protocol and Registration

This systematic review was conducted according to the guidelines of PRISMA (The Preferred Reporting Items for Systematic reviews and Meta-Analyses) [32], and the protocol was registered in the PROSPERO database (no. CRD42023430957).

### 2.2. Search Strategy and Study Selection 

The research framework was defined according to the PICOS (Population, Intervention, Comparison, Outcome, Study design) statement, yielding the following question: “Is ozone treatment clinically effective in arresting or reversing caries progression in primary teeth?”. Therefore, we considered (S) all types of clinical studies (e.g., randomized controlled clinical trials, RCTs, or non-randomized non-controlled clinical trials, non-RCTs, and observational cohort, case–control, or cross-sectional studies, with a prospective or retrospective design) that investigated the efficacy of (I) ozone applications in all formulations compared to (C) no or other interventions (O) in the treatment of caries, including arresting or reversing caries progression (as measured by clinical signs and symptoms and/or microbiological parameters) of (P) primary teeth in children less than or equal to 14 years of age. The PICOS framework outlined also defines the inclusion criteria for this review. Articles related to the use of ozone for preventing the development of caries or for the management of caries in permanent teeth were excluded. The literature search was conducted on the PubMed/MEDLINE, Embase, and Web of Science online databases, on 6 January 2024, using “ozone” AND “caries” as keywords, related MeSH terms, topic terms, and exploded terms on the three databases, respectively. The search strategy, which resulted in the maximum number of results, was chosen among different search lines that were preliminarily tested in order to maximize the retrieval of possibly relevant results. The details of the search strategy are reported in Table 1. No language or date restrictions were applied. Only original research articles were included, while conference proceedings, letters to the editor, commentaries, case reports, reviews, and meta-analyses were not considered.

Handsearching was conducted through backward citation chasing by manually screening the references of the studies included in order to identify possible additional eligible articles. The grey literature was searched using CADTH Grey Matters database by the Canadian Agency for Drugs and Technologies in Health (Ottawa, Canada). All records were imported into Rayyan web app and duplicates were identified and removed through a software algorithm [33]. The screening of titles, abstracts, and full texts for inclusion was performed independently by two authors (FV and TF). Another author (LG) was involved to resolve possible disagreements. 

### 2.3. Data Extraction

The data extraction from the included studies was conducted by one author (FV) and double checked by another author (TF). We considered information related to the first author, date of publication, country, study design, number and characteristics of the participants, type and protocol of ozone treatment, type and protocol of alternative treatment (if any), outcomes of interest and their measures, follow-up duration, and main findings. The extracted data were recorded on Excel datasheets (v. 16.82, Microsoft Office, Redmond, WA, USA) and are presented in tables herein.

### 2.4. Data Analysis

A statistical synthesis in the form of meta-analysis could not be performed due to the limited body of evidence and great heterogeneity of the eligible studies. However, a systematic synthesis and discussion of relevant possible bias and sources of heterogeneity of the available clinical evidence is provided, following relevant reporting guidelines such as SWiM (Synthesis without meta-analysis in systematic reviews) and ENTREQ (The Enhancing Transparency in REporting the synthesis of Qualitative research) [34,35]. 

### 2.5. Risk of Bias Assessment

Risk of bias (RoB) assessment was conducted according to the current versions of Cochrane RoB 2 and ROBINS-I tools [36,37]. For each study, the most appropriate of the two tools was chosen based on the specific study design. The results of the RoB assessment were elaborated with the dedicated Robvis tool [38]. Two authors (FV and TF) conducted the RoB evaluation, with a third author (MV) resolving any discrepancies. Specific criteria applied for this assessment are shown in Supplementary Tables S1 and S2. The study was judged to be at overall low risk of bias if all domains were rated at low risk, to raise some concerns if at least one domain was rated as such, and at high risk of bias if at least one domain was rated as such or 3 or more domains were considered to have some concerns. 

## 3. Results

### 3.1. Study Selection

**Figure 1:**
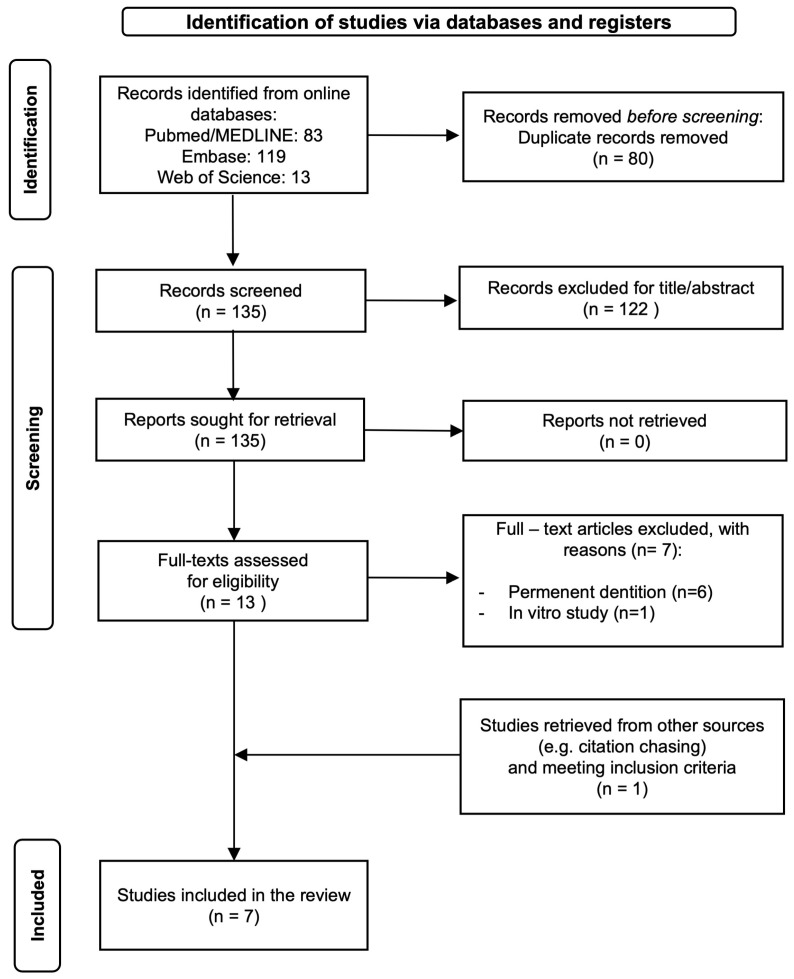
PRISMA 2020 flow chart of study selection process.

Out of 215 potentially relevant records, 135 records were retrieved after duplicates removal. We excluded 122 records based on the screening of titles and abstracts. A further seven papers were excluded due to full-text evaluation (six articles evaluated permanent teeth and one was an in vitro study). One additional eligible paper was retrieved through citation chasing, based on the reference lists of the included studies and the recent reviews. Overall, seven studies were eventually included in this review. The detailed study selection process is shown in Figure 1 as a PRISMA flowchart.

### 3.2. Characteristics of Included Studies

Of the included studies, three were randomized controlled clinical trials [39,40,41], of which one had a split-mouth design [39], two were observational studies with a retrospective cohort design [28,42], one was designed as a non-randomized controlled clinical trial with a split-mouth design [30], and one was a non-controlled clinical trial [43]. 

The studies included in the present review were conducted in Italy, Iraq, Switzerland, Sweden, and Turkey, in a period of time that ranges between 2006 and 2023. The main characteristics of the included studies are summarized in Table 2. A total of 513 carious lesions on primary teeth in 311 children were investigated. The age of the children ranged from 2 to 11 years old. 

The modality and protocols of ozone applications varied greatly across studies. Ozone was applied in gaseous formulation in all studies, either using a vacuum silicone cup when ozone was produced from a pure medical oxygen tank (*n* = 6) or by keeping the handpiece tip perpendicular to the decayed surface at a short distance from it, with ozone being produced from the local environmental oxygen (*n* = 1). The ozone nominal concentration in oxygen was approximately 4.7 g/m^3^ in four studies (in one of which the information was not reported and was obtained from the device specifications), 32 g/m^3^ in one study, and missing information in another study, but according to the manufacturer it should be approximately 0.4 g/m^3^ (i.e., 200 ppm). The duration of each application ranged from 20 to 60 s, for multiple times with different recall protocols.

In controlled studies, ozone treatment was compared either with the standard intervention without ozone application (e.g., manual selective caries excavation), chlorhexidine gel, or fluoride varnish. The outcomes of interest varied between the studies: multiple-item clinical success, including absence of pain and hypersensitivity; caries activity defined by visual inspection, dentin color and hardness, and laser fluorescence; antibacterial activity as indicated by bacterial counts; and parents’ and children’s attitude and treatment-related quality of life. Three studies also looked at the possible presence of adverse effects and found no evidence of these. The cumulative follow-up was of 0, 8, 4, and 12 months, depending on the specific treatment protocol. 

### 3.3. Risk of Bias Assessment

**Figure 2:**
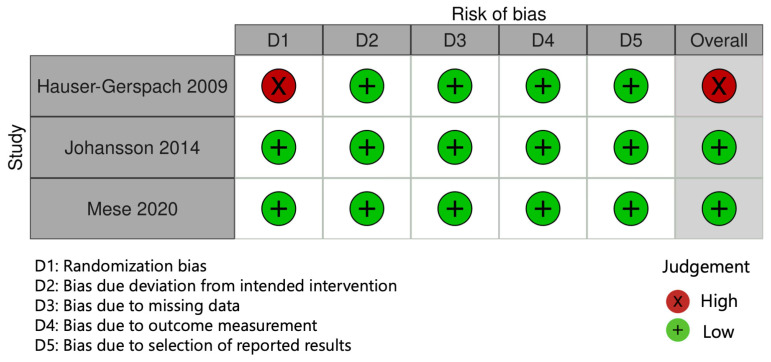
Results of risk of bias assessment for randomized trials, according to the current version of the Cochrane RoB 2 tool [39,40,41].

**Figure 3:**
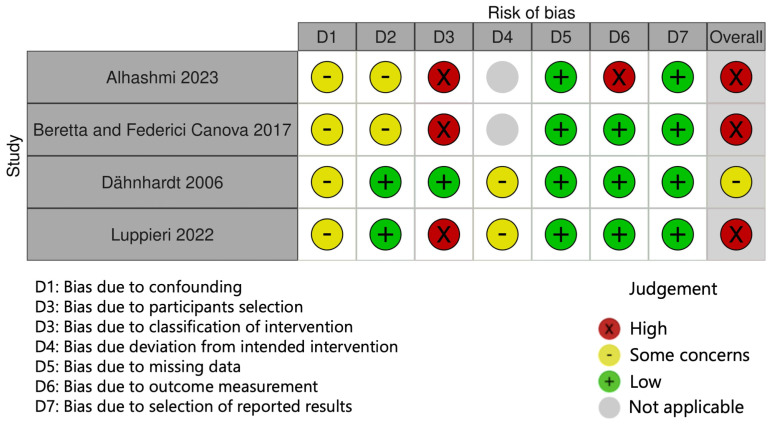
Results of risk of bias assessment for non-randomized studies—of interventions, according to the current version of the Cochrane ROBINS-I tool [28,30,42,43].

The results of the RoB assessment are reported in Figure 2 and Figure 3. Three randomized trials were evaluated using the Rob 2 tool. Among RCTs, one study was judged at high risk in domain D1 due to a lack of detail of the randomization process [40]. Non-randomized clinical studies were assessed using the ROBINS-I tool. Among these, three studies were rated at high risk of bias, either due to the absence of a control group (domain D3) [28,42,43] or to the lack of clearly defined outcome measures (domain D6) [42]. Overall, four studies were judged at “high” risk of bias [28,40,42,43], one of “some concerns” [30], and two were judged at “low” risk of bias [39,41].

### 3.4. Synthesis of Findings

With regards to clinical parameters such as restoration integrity and absence of pain and periapical pathology, one study [28] reported a success rate of 93.62% at 12 months in their non-controlled retrospective study, using gaseous ozone applications for 60 s under vacuum, at a concentration of 32 g/m^3^, after partial removal of carious dentin and followed by composite restoration. Similarly, another study with the same design and procedures [42] reported a clinical success rate of 92.3% at 12 months, using a concentration of 4.7 g/m^3^. The authors of both these retrospective studies also highlighted that most failures occurred after accidental exposure of pulp tissue probably due to the vacuum created by the ozone device that removed the extremely thin residual dentine layer. Another non-controlled study [43] on uncooperative children with Early Childhood Caries (ECC) observed the absence or the regression of hypersensitivity after a cycle of four ozone applications of 60 s (one per week) at low concentrations of approximately 0.4 g/m^3^, without the placement of any restoration. Luppieri et al. [43] also assessed caries activity by evaluating dentin hardness, which significantly improved immediately after ozone application cycles and remained substantially stable throughout the 4-month follow-up. Similar results were achieved by Dähnhardt et al. [30], who conducted a controlled trial on anxious uncooperative children using a total of five gaseous ozone applications of 20 s at a concentration of 4.7 g/m^3^ every two months, after an initial manual excavation. They observed a significant increase in dentin hardness throughout the follow-up of 8 months, whereas no significant changes were detected in the control group. Mese et al. [41] compared ozone applications (4.7 g/m^3^, for 60 s) with chlorhexidine 2% solution and no additional treatment, after selective caries removal and followed by a temporary restoration, and reported an improvement in dentin features, including hardness and color, with no significant differences among treatment groups at the 4-month follow up. Visual inspection according to Ekstrand et al. [44] was also evaluated as a measure of caries activity and severity by Johansson et al. [39], who compared trimestral ozone applications at 4.7 g/m^3^ for 40 s, with fluoride varnish applications (22,600 ppm NaF), in a two-phase study for the management of cavitated and non-cavitated lesions. The authors concluded that throughout the overall follow-up of 12 months, neither ozone nor fluoride varnish stopped or reversed the progression of caries in cavitated lesions, while non-cavitated lesions showed slight or no progression similarly in both treatment groups; similarly, caries activity measured through laser fluorescence showed no significant differences between groups [39]. On the other hand, Dähnhardt et al. [30] observed a greater improvement with laser fluorescence, yet this was not significantly different, in the ozone group as compared to manual selective caries excavation alone.

With regard to the bacterial load within carious lesions, Mese et al. [41] reported a reduction in the total number of microorganisms in all groups 4 months after treatment, with reduction percentages greater in the chlorhexidine group, followed by the ozone group compared to the control group; the total bacterial count showed no significant differences among groups, while concerning the decrease in specific *S. mutans* and Lactobacilli counts, immediately after application chlorhexidine was reported to be more effective than ozone. Conversely, a study by Hauser-Gerspach et al. [40] found no remarkable reduction in total bacterial count either with gaseous ozone (4.7 g/m^3^, for 30 s) or chlorhexidine 1% gel applications, on both excavated and non-excavated carious lesions. Luppieri et al. [43], on the other hand, reported strong evidence of ozone’s antibacterial effectiveness against *S. mutans*, yet in a non-controlled study setting. 

The two studies additionally evaluating the domain of patient-reported outcomes indicated promising results. Luppieri et al. [43] administered the validated Early Childhood Oral Health Impact Scale (ECOHIS) questionnaire to parents and recorded a marked improvement in the quality of life of patients and families in relation to ECC. Dähnhardt et al. [30] investigated parents’ and children’s attitude towards ozone before and after treatment through specifically designed questionnaires administered to the parents, finding that approximately all parents were happy with the treatment and management of caries with ozone led to the reduction in dental anxiety for 93% of the children. In addition, no side effects were reported by patients [30,39,40].

## 4. Discussion

The intended goal of this review was to investigate the effectiveness of ozone treatment in the management of carious lesions in primary teeth according to all available clinical evidence. Unfortunately, a meta-analysis could not be conducted due to the limited body of evidence and potential methodological limitations of the included studies. In particular, the great heterogeneity of study designs, intervention protocols, and outcomes considered has led to inconsistent overall results, highlighting the need for more standardized research in this field. Nevertheless, overall promising results emerged from this review. These can be explained by the beneficial effects of ozone on dental tissue, which include remineralizing and anti-bacterial properties. Also, although the effect of ozone on dentin is less studied, it has been reported that in addition to improving the diffusion of salivary ions to the surface of degraded dentin, ozone can neutralize acidic proteins produced by cariogenic bacteria, which constitute the osmotic stimulus responsible for the movement of fluids in the dentin tubules that causes hypersensitivity [45,46]. This can explain the positive impact on caries-related symptoms, such as pain or hypersensitivity, reported by some studies [28,43,47]. 

A common limitation to some studies was the uncontrolled design [28,42,43], where ozone treatment was found to be effective but was not compared with other available approaches (e.g., complete or selective caries removal, including rotary instruments or manual excavation, chemo-mechanical removal, restorative procedures, remineralization procedures) [7,48]. Dähnhardt et al. [30] used ozone application as an additional tool and only compared it to manual excavation alone. Nevertheless, these study settings were adopted in clinical situations where lack of cooperation due to young age, dental fear, or otherwise complex and time-consuming intervention (e.g., pulpotomy) would have prevented the use of other traditional management strategies [30,42,43]. Where a comparison with other interventions was provided, it was based on the specific outcome, and it was either with other antimicrobial agents (e.g., chlorhexidine) or a remineralizing agent such as fluoride varnish [39,40,41]. Overall, these studies generally found no remarkable differences in clinical and antimicrobial outcomes between ozone treatment and positive controls over the entire follow-up period, while a slight antibacterial superiority for chlorhexidine was found at some intermediate time points [41]. It should also be noted that these controlled trials did not address intervention-related quality of life outcomes in children or the acceptance of treatments [39,40,41]. In fact, it is well known that organoleptic characteristics such as the taste of some dental products and oral antiseptics (e.g., topical and local anesthetics, chlorhexidine, sodium chloride gels, etc.) can cause significant discomfort in young patients, sometimes leading to partial or total loss of compliance and refusal of further treatments [49,50]. Mese et al. [41], unlike two other controlled studies [30,39], though being rated at low RoB, did not adopt a split-mouth design, while Hauser-Gerspach et al. [40] did adopt a split-mouth design, but only with regard to the excavation versus non-excavation procedure, and not specifically for the allocation of the ozone versus control treatment [40]. This choice and the related risk of bias might have affected their findings. Overall, ozone treatment must be considered as a component of a multi-tool oral care strategy, which includes individual factors such as a healthy and anti-carious dietary regimen, the use of fluoride-containing and remineralizing products, and the implementation of appropriate dental care programs and oral hygiene measures [51,52,53]. These factors can influence the risk of caries, the clinical evolution of carious lesions, and the effectiveness over time of all management interventions. A split-mouth design, where one of two treatments is randomly assigned to either one or the other corresponding tooth in the same patient, can be a valid solution to overcome such issue, as it is expected to remove most inter-individual variability [54]. Interestingly, among the studies rated at low risk of bias, the one adopting a split-mouth design found that ozone treatment of non-cavitated lesions yielded results comparable to fluoride varnish application in terms of fluorescence values and visual inspection index, with slight or no progression similar in both groups [39]. This suggests that ozone may be a valid alternative to professionally applied topical fluoride products in very young or uncooperative patients, where accidental ingestion may occur [55]. It should be considered that early excessive exposure to fluoride has been associated, albeit controversially, with a number of potential adverse health effects and its use may be opposed by some parents or caregivers [56,57,58,59,60]. 

Additionally, the caries diagnostic criteria defining the inclusion of patients or the unit of analyses (i.e., pair of teeth, tooth) varied widely across studies and were mostly based on clinical subjective evaluations, such as caries extension or visual appearance according to the Ekstrand criteria [44]. Such criteria often lacked a precise definition and objectivation of “extensive” or “deep” carious lesion, thus possibly impacting the clinical baseline at which intervention was administered [28,30,40,41,42]. This is, however, a common shortcoming of studies investigating minimally invasive treatments in cavitated carious lesions of young and poorly compliant patients, due to the difficulties in applying more rigorous and objective diagnostic and case-definition methods, in addition to the current limited availability of biological tools to assess caries activity [61,62]. Consequently, outcome assessment could have been another source of heterogeneity across studies. Outcomes considered in the studies belonged in fact to different domains, i.e., clinical, microbiological, and treatment acceptance. In addition, different assessment methods were sometimes chosen for the same domain. A specific definition of “clinical success” was not provided in one study [42]. Caries activity was monitored according to dentin consistency and/or color subjective evaluation, whether using validated [39] and non-validated scales [30,41,43]. Two studies additionally used laser fluorescence measurement to monitor caries activity, which is a validated method expected to provide a more accurate and objective assessment [30,39]. However, many trials and systematic reviews have found great variability and poor reliability of laser fluorescence, under different clinical conditions [63,64,65]. Lastly, discrepancy in ozone treatment protocols was a major source of heterogeneity that hampers reliable comparisons among the included studies. Ozone was always used in gaseous formulation. Medical ozone generators can produce ozone through an open system, used in proximity with continuous suction or through a sealed suction system using silicon cups. Only one of the included studies used an open-system device [43]. A sealed suction system is usually considered a safer option, allowing for higher and more effective ozone concentrations to be delivered, while preventing possible adverse effects that may occur upon inhalation [28,51]. Information regarding ozone concentration and flow rate were not always clearly reported and in two cases they were drawn from manufacturers’ specifications [42,43]. It is reported that ozone concentrations as low as 0.1 to 20 ppm (i.e., approximately 2 × 10^−4^ to 0.04 g/m^3^) in a gaseous mixture are already toxic for bacteria [66,67]. Ozone concentration was significantly higher than this threshold in all included studies: In one study, it was 32 g/m^3^ (i.e., 14,000 ppm) [28], while in most studies it was 2100 ppm (i.e., 4.7 g/m^3^) [30,39,40,41,42]. In the study by Luppieri et al. [43], the concentration was remarkably lower (i.e., 200 ppm, approximately 0.4 g/m^3^) and ozone was applied in a non-vacuum setting, as mentioned earlier, making it difficult to infer the effective concentration reaching the tooth surface. Overall, this significant variability may have affected the treatment outcomes. Great discrepancies also emerged in the duration of applications (from 20 s to 60 s), which were performed in one or multiple sessions. Moreover, comparisons between different ozone treatment protocols were not performed, thus not allowing a conclusive synthesis of the results. 

The main limitations of this review are related to the limited quantity and quality of the available evidence, the different study designs and not standardized methods, and the relatively small sample size characterizing the large majority of these studies. These limitations and inconsistencies of the studies reduce the robustness of the overall analysis here performed, making it difficult to draw consistent conclusions and suggesting major research gaps to be addressed by further well-designed and more powerful studies. However, the overall information gathered in this review allows a systematic assessment of the available evidence on the performances of ozone in the treatment of caries of primary dentition, suggesting its potential usefulness in the management of pediatric caries, especially in very young and poorly compliant patients, as a minimally invasive approach that is part of modern pediatric dentistry. 

The occurrence of potential adverse effects due to inhalation toxicity must be considered, as well as a number of general contraindications to ozone therapy, such as recent myocardial infarction, hyperthyroidism, acute alcohol intoxication, severe anemia, thrombocytopenia, active bleeding, and pregnancy [68]. However, with careful use of modern technology and adherence to manufacturers’ recommendations, ozone therapy appears to be free of side effects [69]. 

Specific guidelines and recommendations on the use of ozone for the treatment of caries are not currently available due to the lack of robust and standardized evidence. Based on the results of the present review, the authors hypothesize that ozone applications may be useful for the treatment of caries in primary dentition when other evidence-based conventional or minimally invasive approaches are not applicable, due to limited cooperation or specific contraindications. When using gaseous ozone delivered by open-system devices, continuous suction should always be used in close proximity, otherwise a sealed suction system should be preferred. If neither is available, ozonated water may be a safer option. In the case of very young patients, ozone application may also serve as a transitional intervention that can help children gain confidence and comfort and become accustomed to dental treatment. In any case, ozone application should be implemented within a systematic oral care program that includes follow-up and education on individual oral care and dietary habits.

## 5. Conclusions

Although no conclusive support emerged for recommending the use of ozone over other conventional strategies, and despite some inconsistencies, the overall evidence suggests that ozone application may have beneficial effects, regardless of the protocol applied, being equally or more effective than other interventions, when comparisons were available, in relation to most clinical outcomes, and equally or slightly less effective than chlorhexidine in relation to antibacterial action. Additionally, ozone application appeared to be a simple and pain-free technique, and it was not associated with adverse effects. These features may contribute to reducing parent and child anxiety and improving their compliance, and these aspects may have a considerable role in the choice of treatment strategy.

## Figures and Tables

**Table 1:** Details of literature search strategies in online databases.

Database	Search Strategy
PubMed	(ozone[MH] OR ozone[tiab]) AND (“Dental Caries”[Mesh] OR “Dental Caries”[tiab]) AND humans[MH]
Embase	(‘ozone’/exp OR ‘ozone’) AND ‘dental caries’/exp AND ‘human’/exp
Web of Science	TS = (ozone) AND (TS = dental caries OR TI = dental caries OR AB = dental caries) AND TS = (human)

**Table 2:** Characteristics of the studies included in the review, [28,30,39,40,41,42,43].

Reference	Study Design	Country	Participants	Ozone Treatment	Comparisons	Outcomes of Interest	Follow-up	Main Results
Alhashmi et al., 2023	Retrospective cohort study	Iraq	35 children (M:F = 19:16) Age range: 3–6 years Unit of analysis: extensive cavitated carious lesions of primary molars (*n* = 52)	Gaseous ozone, applied with single-patient silicone cup; applied after partial removal of carious dentin using round carbide burs, followed by composite restoration with rubber dam isolation. 4.7 g/m^3^ †, under vacuum, 60 s HealOzone ^a^	n/a	Clinical success (not defined)	3, 6, 12 months	At 12 months, clinical success was observed in 48/52 treated teeth (92.3%)
Beretta and Federici Canova; 2017	Retrospective cohort study	Italy	50 children (M:F = 28:22) Mean age: 5.8 ± 1.7 years Unit of analysis: extensive cavitated carious lesions of primary molars (*n* = 94)	Gaseous ozone, applied with single-patient silicone cup; applied after partial removal of carious dentin using round carbide burs, followed by composite restoration with rubber dam isolation. 32 g/m^3^, under vacuum, 60 s HealOzone X4 ^a^	n/a	Clinical success as defined by the presence of all the following items: Restoration still in place. Absence of marginal microleakage. Absence of the restoration fractures. Presence of an interproximal contact (for Class II cavities). Absence of discoloration. Absence of pain Absence of pus or fistulas.	3, 6, 12 months	At 12 months, clinical success was observed in 88/94 treated teeth (93.62%)
Dähnhardt et al., 2006	Non-randomized controlled clinical trial (split-mouth)	Switzerland	28 children (M:F = 9:23) Age range: 3–11 years Mean age 5.96 ± 2.36 years Unit of analysis: open single-surface carious lesion (*n* = 82)	Gaseous ozone applied after manual excavation to leathery consistency 4.7 g/m^3^, under vacuum, 20 s, a total of 5 times in 2-months intervals HealOzone ^a^	Manual excavation to leathery consistency	Dentin hardness; Laser Fluorescence—LF (DIAGNOdent ^a^); Parental and children attitude (questionnaire).	0, 2, 4, 6, 8 months	Dentin hardness markedly increased in ozone group over time; no changes were observed in control group. LF values improved more in ozone group than in control group (non significant difference between groups). 93% of children reduced their dental anxiety following ozone treatment. No adverse effects were observed
Hauser-Gerspach et al., 2009	Randomized controlled clinical trial	Switzerland	40 children (M:F = 13:7) Age range: 2–8 years Mean age 5.1 years Unit of analysis: open single-surface carious lesion (*n* = 80)	Gaseous ozone applied on excavated/non excavated lesions 4.7 g/m^3^ (615 mL/min flow rate) under vacuum for 30 s HealOzone ^a^	Chlorhexidine 1% gel applied on excavated/non excavated lesions for 30 s	Total bacterial count (CFU)	None (suitable restoration was placed after the experimental treatment)	Ozone application as well as 1% chlorhexidine gel application did not significantly reduce microorganisms count (CFU). The removal of decayed tissue showed no significant effect either. No adverse effects were observed
Johansson et al., 2014	Randomized controlled clinical trial (split-mouth) 2 phases	Sweden	Total sample: 33 children; 50 pairs of carious lesions First phase: 11 children (M:F = 4:7) Mean age: 4.8 years Age range 3–6 years Unit of analysis: pairs of primary molars with cavitated caries lesions (visual inspection- VI score ≤ 3) (*n* = 15) Second phase: 22 children (M:F = 14:8) Mean age: 4.5 years Age range: 4–8 years Unit of analysis: pairs of primary molars with non-cavitated caries lesions (VI score ≤ 2a) (*n* = 35)	Gaseous ozone 4.7 g/m^3^ (615 mL/min flow rate), under vacuum, for 40 s at baseline, 3, 6, and 9 months or until failure (i.e., VI score 4) HealOzone ^a^	Fluoride varnish 22,600 ppm NaF, applied in a thin layer with a micro-brush, at baseline, 3, 6, and 9 months or until failure (i.e., VI score 4) Duraphat^® b^	Visual Inspection (VI) according to Ekstrand score [44] Laser fluorescence (LF)- DIAGNOdent ^a^	Baseline, 3, 6, 9, and 12 months or until failure (i.e., VI score 4) indicating necessity of an operative treatment.	Phase 1: In the 15 pairs VI ≤ 3 lesions, all of the lesions failed during the 12-month period (8 treated with ozone and 9 with fluoride). Median baseline LF values in the VI ≤ 3 group were 76 for ozone and 69 for fluoride group. At 12 months, they were 57 and 58, respectively. Phase 2: In the 35 pairs VI ≤ 2a lesions, one lesion failed (treated with ozone) after 12 months. Median baseline LF values were 21 and 19 for ozone and fluoride, respectively. At 12 months, median LF values were 15 for ozone group and 17.5 for fluoride–varnish group. No difference in LF values was observed over time within and between groups. In cavitated lesions, neither ozone nor fluoride varnish treatments stopped the progression of caries. Non-cavitated lesions showed slight or no progression following both treatments. No adverse effects were observed
Luppieri et al., 2022	Non-controlled clinical trial	Italy	20 children with ECC (M:F = 9:11) Age range: 3–5 years Mean age 4.5 Unit of analysis: decayed tooth (*n* = 20; 9 molars, 8 incisors, and 1 canine)	Gaseous ozone; application on cleaned tooth (rotating low-speed brush) using ozone intensity program n.6 (of 10), 0.4 g/m^3^ †, for 60 s, keeping the probe’s tip in continuous motion and perpendicular to the decayed surface at 1 mm from it; one application per week, for four consecutive weeks. OzoneDTA^® c^	n/a	Dentin compactness Dentin hypersensitivity to thermal stimuli (air syringe spray) according to the Wong–Baker Faces Pain Rating Scale (WBFPRS) Salivary Bacterial Count (*Streptococcus mutans*) at T0 and T1 Patients’ and families’ quality of life—Early Childhood Oral Health Impact Scale (ECOHIS) at T0 and T1	T0 = baseline; T1 = after the 4-sessions ozone treatment cycle; T2 = 1 month from T1; T3 = 2 months from T1, T4 = 3 months from T1.	Affected dentin compactness significantly improved from T0 to T1. A mild improvement (not statistically significant) was detected at the other time points. 2 children reported hypersensitivity/pain at baseline, with a mean reported value of 3.5 according to the WBFPRS. At T1, none of the patients reported dentin hypersensitivity; no further changes were observed at later follow-ups. *S. mutans* count (*n* = 11) decreased significantly from T0 to T1. 8 of the 9 patients that were positive at T0 became negative at T1, while no changes were detected for the negative ones. The mean ECOHIS score was 9.4 at T0 and was 5.9 at T1
Mese et al., 2020	Randomized controlled clinical trial	Turkey	105 children (M:F = 46:59) Age range: 6–10 Unit of analysis: deep caries lesion of lower primary molars (*n* = 105)	Gaseous ozone (*n* = 35), applied after partial removal of carious dentin (stepwise excavation), 4.7 g/m^3^, under vacuum, for 60 s HealOzone ^a^	Partial removal of carious dentin (stepwise excavation) followed by: I.CHX 2% solution (*n* = 35) applied once for 60 s using a brush II. Control—no disinfectant treatment (*n* = 35) In all 3 groups, Ca(OH)_2_ liner was placed on the remaining carious dentin and the tooth was temporarily restored.	Clinical evaluation of dentin color, consistency, and humidity Total and specific bacterial counts (CFUs) for *Streptococcus mutans* and *Lactobacillus* spp., on dentine sample	Immediately after treatment and after 4 months (at replacement of temporary restoration) T0: immediately after partial removal of necrotic carious dentin T1: immediately after applying the disinfection procedure T2: 4-month follow-up (immediately after the removal of the temporary restoration) T3: At the second appointment, after complete removal of the remaining carious dentin.	Total incidence of pulp exposure was similar among groups (3.03% in the control group, 3.125% in the CHX group, and 2.85% in the ozone group). Dentin became gradually dryer and harder in all the groups, and there was no significant difference among the groups both between T0 and T2, and T0 and T3. Dentin color became darker at the second appointment in the CHX and ozone groups, with significant differences as compared to control group, but no significant difference among treatment groups. The reduction percentages of the total number of microorganism species were significantly higher in the CHX and ozone groups compared to the control group. CHX resulted in significantly higher reduction than ozone between T0-T1 and T0-T2. Total CFU showed no significant difference among groups between T0 and T3. With regards to *S. mutans* and *Lactobacillus* spp. CFUs, CHX was significantly more effective than ozone between T0–T1

Abbreviations: CFU: colony-forming units; CHX: chlorhexidine digluconate. † This information was not reported in the original article and was obtained from the publicly available technical specifications of the device; ^a^ = KaVo Dental GmbH, Germany; ^b^ = Colgate, NY, USA; ^c^ = Sweden & Martina, Italy.

## Data Availability

All data associated with this study are included in the article and Supplementary Material.

## Supplementary Materials

The following supporting information can be downloaded at: https://www.mdpi.com/article/10.3390/dj12030069/s1, Table S1: Criteria adopted for risk of bias assessment using the current version of the Cochrane RoB 2 for randomized trials; Table S2: Criteria adopted for risk of bias assessment using the current version of the Cochrane ROBINS-I tool (Risk of Bias in Non-randomized Studies—of Interventions).

## References

[1] GBD 2017 Disease and Injury Incidence and Prevalence Collaborators (2018). Global, Regional, and National Incidence, Prevalence, and Years Lived with Disability for 354 Diseases and Injuries for 195 Countries and Territories, 1990–2017: A Systematic Analysis for the Global Burden of Disease Study 2017. Lancet.

[2] Kassebaum N.J., Bernabé E., Dahiya M., Bhandari B., Murray C.J.L., Marcenes W. (2015). Global Burden of Untreated Caries: A Systematic Review and Metaregression. J. Dent. Res..

[3] Skeie M.S., Sen A., Dahllöf G., Fagerhaug T.N., Høvik H., Klock K.S. (2022). Dental Caries at Enamel and Dentine Level among European Adolescents—A Systematic Review and Meta-Analysis. BMC Oral Health.

[4] Benelli K.d.R.G., Chaffee B.W., Kramer P.F., Knorst J.K., Ardenghi T.M., Feldens C.A. (2022). Pattern of Caries Lesions and Oral Health-related Quality of Life throughout Early Childhood: A Birth Cohort Study. Eur. J. Oral Sci..

[5] Zaror C., Matamala-Santander A., Ferrer M., Rivera-Mendoza F., Espinoza-Espinoza G., Martínez-Zapata M.J. (2022). Impact of Early Childhood Caries on Oral Health-related Quality of Life: A Systematic Review and Meta-analysis. Int. J. Dent. Hyg..

[6] Duggal M., Gizani S., Albadri S., Krämer N., Stratigaki E., Tong H.J., Seremidi K., Kloukos D., BaniHani A., Santamaría R.M. (2022). Best Clinical Practice Guidance for Treating Deep Carious Lesions in Primary Teeth: An EAPD Policy Document. Eur. Arch. Paediatr. Dent..

[7] Leal S.C. (2014). Minimal Intervention Dentistry in the Management of the Paediatric Patient. Br. Dent. J..

[8] Sabbagh H.J., Albeladi N.H., Altabsh N.Z., Bamashmous N.O. (2023). Risk Factors Associated with Children Receiving Treatment at Emergency Dental Clinics: A Case-Control Study. Int. J. Environ. Res. Public. Health.

[9] Dahlander A., Soares F., Grindefjord M., Dahllöf G. (2019). Factors Associated with Dental Fear and Anxiety in Children Aged 7 to 9 Years. Dent. J..

[10] Desai H., Stewart C.A., Finer Y. (2021). Minimally Invasive Therapies for the Management of Dental Caries—A Literature Review. Dent. J..

[11] Innes N.P.T., Evans D.J.P. (2014). Managing Caries in Primary Teeth. BDJ Team.

[12] Santamaria R.M., Innes N.P.T., Machiulskiene V., Evans D.J.P., Splieth C.H. (2014). Caries Management Strategies for Primary Molars: 1-Yr Randomized Control Trial Results. J. Dent. Res..

[13] Conti G., Veneri F., Amadori F., Garzoni A., Majorana A., Bardellini E. (2023). Evaluation of Antibacterial Activity of a Bioactive Restorative Material Versus a Glass-Ionomer Cement on Streptococcus Mutans: In-Vitro Study. Dent. J..

[14] Veneri F., Bardellini E., Amadori F., Gobbi E., Belotti R., Majorana A. (2020). Antibacterial Activity of New Hydrophilic Sealants: In Vitro Study. J. Indian. Soc. Pedod. Prev. Dent..

[15] Gupta N., Chowdhary N., Reddy V.R., Nk K., Peddi R., Kumar M. (2022). Evaluation of Caries Removal Efficacy Using BRIX 3000 and Atraumatic Restorative Treatment in Primary Molars: A Clinical Comparative Study. J. Contemp. Dent. Pract..

[16] Alkhouli M.M., Al Nesser S.F., Bshara N.G., AlMidani A.N., Comisi J.C. (2020). Comparing the Efficacies of Two Chemo-Mechanical Caries Removal Agents (2.25% Sodium Hypochlorite Gel and Brix 3000), in Caries Removal and Patient Cooperation: A Randomized Controlled Clinical Trial. J. Dent..

[17] Contreras V., Toro M.J., Elías-Boneta A.R., Encarnación-Burgos A. (2017). Effectiveness of Silver Diamine Fluoride in Caries Prevention and Arrest: A Systematic Literature Review. Gen. Dent..

[18] Splieth C.H., Banerjee A., Bottenberg P., Breschi L., Campus G., Ekstrand K.R., Giacaman R.A., Haak R., Hannig M., Hickel R. (2020). How to Intervene in the Caries Process in Children: A Joint ORCA and EFCD Expert Delphi Consensus Statement. Caries Res..

[19] Al-Yaseen W., Seifo N., Bhatia S., Innes N. (2021). When Less Is More: Minimally Invasive, Evidence-Based Treatments for Dentine Caries in Primary Teeth—The Hall Technique and Silver Diamine Fluoride. Prim. Dent. J..

[20] Santos G.M., Pacheco R.L., Bussadori S.K., Santos E.M., Riera R., De Oliveira Cruz Latorraca C., Mota P., Benavent Caldas Bellotto E.F., Martimbianco A.L.C. (2020). Effectiveness and Safety of Ozone Therapy in Dental Caries Treatment: Systematic Review and Meta-Analysis. J. Evid. Based Dent. Pract..

[21] Costa T., Linhares D., Ribeiro da Silva M., Neves N. (2018). Ozone Therapy for Low Back Pain. A Systematic Review. Acta Reumatol. Port..

[22] Veneri F., Bardellini E., Amadori F., Conti G., Majorana A. (2020). Efficacy of Ozonized Water for the Treatment of Erosive Oral Lichen Planus: A Randomized Controlled Study. Med. Oral Patol. Oral Cir. Bucal.

[23] Di Fede O., Del Gaizo C., Panzarella V., La Mantia G., Tozzo P., Di Grigoli A., Lo Casto A., Mauceri R., Campisi G. (2022). Ozone Infiltration for Osteonecrosis of the Jaw Therapy: A Case Series. J. Clin. Med..

[24] Oliveira Modena D.A., de Castro Ferreira R., Froes P.M., Rocha K.C. (2022). Ozone Therapy for Dermatological Conditions: A Systematic Review. J. Clin. Aesthet. Dermatol..

[25] Rapone B., Ferrara E., Santacroce L., Topi S., Gnoni A., Dipalma G., Mancini A., Di Domenico M., Tartaglia G.M., Scarano A. (2022). The Gaseous Ozone Therapy as a Promising Antiseptic Adjuvant of Periodontal Treatment: A Randomized Controlled Clinical Trial. Int. J. Environ. Res. Public Health.

[26] Shang W., Wang Y., Wang G., Han D. (2023). Benefits of Ozone on Mortality in Patients with COVID-19: A Systematic Review and Meta-Analysis. Complement. Ther. Med..

[27] Bardellini E., Amadori F., Veneri F., Conti G., Majorana A. (2020). Coronavirus Disease-2019 and Dental Practice: A Project on the Use of Ozonized Water in the Water Circuit of the Dental Armchair. Stomatologija.

[28] Beretta M., Federici Canova F. (2017). A New Method for Deep Caries Treatment in Primary Teeth Using Ozone: A Retrospective Study. Eur. J. Paediatr. Dent..

[29] Celiberti P., Pazera P., Lussi A. (2006). The Impact of Ozone Treatment on Enamel Physical Properties. Am. J. Dent..

[30] Dähnhardt J.E., Jaeggi T., Lussi A. (2006). Treating Open Carious Lesions in Anxious Children with Ozone. A Prospective Controlled Clinical Study. Am. J. Dent..

[31] Crystal Y.O., Marghalani A.A., Ureles S.D., Wright J.T., Sulyanto R., Divaris K., Fontana M., Graham L. (2017). Use of Silver Diamine Fluoride for Dental Caries Management in Children and Adolescents, Including Those with Special Health Care Needs. Pediatr. Dent..

[32] Page M.J., McKenzie J.E., Bossuyt P.M., Boutron I., Hoffmann T.C., Mulrow C.D., Shamseer L., Tetzlaff J.M., Akl E.A., Brennan S.E. (2021). The PRISMA 2020 Statement: An Updated Guideline for Reporting Systematic Reviews. BMJ.

[33] Ouzzani M., Hammady H., Fedorowicz Z., Elmagarmid A. (2016). Rayyan—A Web and Mobile App for Systematic Reviews. Syst. Rev..

[34] Tong A., Flemming K., McInnes E., Oliver S., Craig J. (2012). Enhancing Transparency in Reporting the Synthesis of Qualitative Research: ENTREQ. BMC Med. Res. Methodol..

[35] Campbell M., McKenzie J.E., Sowden A., Katikireddi S.V., Brennan S.E., Ellis S., Hartmann-Boyce J., Ryan R., Shepperd S., Thomas J. (2020). Synthesis without Meta-Analysis (SWiM) in Systematic Reviews: Reporting Guideline. BMJ.

[36] Sterne J.A.C., Savović J., Page M.J., Elbers R.G., Blencowe N.S., Boutron I., Cates C.J., Cheng H.-Y., Corbett M.S., Eldridge S.M. (2019). RoB 2: A Revised Tool for Assessing Risk of Bias in Randomised Trials. BMJ.

[37] Sterne J.A., Hernán M.A., Reeves B.C., Savović J., Berkman N.D., Viswanathan M., Henry D., Altman D.G., Ansari M.T., Boutron I. (2016). ROBINS-I: A Tool for Assessing Risk of Bias in Non-Randomised Studies of Interventions. BMJ.

[38] McGuinness L.A., Higgins J.P.T. (2021). Risk-of-bias VISualization (Robvis): An R Package and Shiny Web App for Visualizing Risk-of-bias Assessments. Res. Synth. Methods.

[39] Johansson E., Van Dijken J.W.V., Karlsson L., Andersson-Wenckert I. (2014). Treatment Effect of Ozone and Fluoride Varnish Application on Occlusal Caries in Primary Molars: A 12-Month Study. Clin. Oral Investig..

[40] Hauser-Gerspach I., Pfäffli-Savtchenko V., Dähnhardt J.E., Meyer J., Lussi A. (2009). Comparison of the Immediate Effects of Gaseous Ozone and Chlorhexidine Gel on Bacteria in Cavitated Carious Lesions in Children In Vivo. Clin. Oral Investig..

[41] Mese M., Tok Y.T., Kaya S., Akcay M. (2020). Influence of Ozone Application in the Stepwise Excavation of Primary Molars: A Randomized Clinical Trial. Clin. Oral Investig..

[42] Alhashmi A.A., Mohammed R.A.K., Hasan M.S. (2023). An Innovative Technique for Treating Severe Caries in Primary Teeth with Ozone: A Retrospective Investigation. J. Pharm. Negat. Results.

[43] Luppieri V., Manfra A., Ronfani L., Chermetz M., Cadenaro M. (2022). Ozone Therapy for Early Childhood Caries (ECC) Treatment: An In Vivo Prospective Study. Appl. Sci..

[44] Ekstrand K.R., Ricketts D.N.J., Kidd E.A.M., Qvist V., Schou S. (1998). Detection, Diagnosing, Monitoring and Logical Treatment of Occlusal Caries in Relation to Lesion Activity and Severity: An in Vivo Examination with Histological Validation. Caries Res..

[45] Raafat Abdelaziz R., Mosallam R.S., Yousry M.M. (2011). Tubular Occlusion of Simulated Hypersensitive Dentin by the Combined Use of Ozone and Desensitizing Agents. Acta Odontol. Scand..

[46] Karlsson L., Kjaeldgaard M. (2017). Ozone Treatment on Dentin Hypersensitivity Surfaces—A Pilot Study. Open Dent. J..

[47] Rupel K., Ottaviani G., Bogdan Preda M.T., Poropat A., Gobbo M., DI Lenarda R., Biasotto M. (2023). Ozone Treatment Combined with Sodium Fluoride for the Treatment of Dentin Hypersensitivity: An Exploratory Study. Minerva Dent. Oral Sci..

[48] Meyfarth S., Cassano K., Warol F., Santos M.D.D., Scarparo A. (2020). A New Efficient Agent to Chemo-Mechanical Caries Removal. Rev. Bras. Odontol..

[49] Cardoso M., Coelho A., Lima R., Amaro I., Paula A., Marto C.M., Sousa J., Spagnuolo G., Marques Ferreira M., Carrilho E. (2020). Efficacy and Patient’s Acceptance of Alternative Methods for Caries Removal—A Systematic Review. JCM.

[50] Sajadi F.S., Rostamizadeh M., Hasheminejad J., Hasheminejad N., Borna R., Bazrafshani M. (2021). Effect of Chlorhexidine, Fluoride and Green Tea Oral Gel on Pediatric Salivary Cariogenic Bacteria: A Clinical Trial Study. Int. J. Pediatr..

[51] D’Amario M., Di Carlo M., Natale S.M., Memè L., Marzo G., Matarazzo G., Capogreco M. (2022). Application of Ozone Therapy in Paediatric Dentistry. Appl. Sci..

[52] Veneri F., Vinceti S., Filippini T. (2024). Fluoride and Caries Prevention: A Scoping Review of Public Health Policies. Ann. Ig..

[53] Vinceti S.R., Veneri F., Filippini T. (2024). Water Fluoridation between Public Health and Public Law: An Assessment of Regulations across Countries and Their Preventive Medicine Implications. Ann. Ig..

[54] Lesaffre E., Philstrom B., Needleman I., Worthington H. (2009). The Design and Analysis of Split-Mouth Studies: What Statisticians and Clinicians Should Know: THE SPLIT-MOUTH DESIGN. Statist. Med..

[55] (2023). American Academy of Pediatric Dentistry Fluoride Therapy. The Reference Manual of Pediatric Dentistry.

[56] Veneri F., Vinceti M., Generali L., Giannone M.E., Mazzoleni E., Birnbaum L.S., Consolo U., Filippini T. (2023). Fluoride Exposure and Cognitive Neurodevelopment: Systematic Review and Dose-Response Meta-Analysis. Environ. Res..

[57] Iamandii I., De Pasquale L., Giannone M.E., Veneri F., Generali L., Consolo U., Birnbaum L.S., Castenmiller J., Halldorsson T.I., Filippini T. (2024). Does Fluoride Exposure Affect Thyroid Function? A Systematic Review and Dose-Response Meta-Analysis. Environ. Res..

[58] Veneri F., Iamandii I., Vinceti M., Birnbaum L.S., Generali L., Consolo U., Filippini T. (2023). Fluoride Exposure and Skeletal Fluorosis: A Systematic Review and Dose-Response Meta-Analysis. Curr. Envir Health Rep..

[59] Fiore G., Veneri F., Di Lorenzo R.D., Generali L., Vinceti M., Filippini T. (2023). Fluoride Exposure and ADHD: A Systematic Review of Epidemiological Studies. Medicina.

[60] Fernandes I.C., Forte F.D.S., Sampaio F.C. (2021). Molar-Incisor Hypomineralization (MIH), Dental Fluorosis, and Caries in Rural Areas with Different Fluoride Levels in the Drinking Water. Int. J. Paediatr. Dent..

[61] Ismail A.I., Sohn W. (1999). A Systematic Review of Clinical Diagnostic Criteria of Early Childhood Caries. J. Public Health Dent..

[62] Ismail A.I., Pitts N.B., Tellez M. (2015). Authors of the International Caries Classification and Management System (ICCMS) The International Caries Classification and Management System (ICCMS^TM^) An Example of a Caries Management Pathway. BMC Oral Health.

[63] Karlsson L., Johansson E., Tranæus S. (2009). Validity and Reliability of Laser-Induced Fluorescence Measurements on Carious Root Surfaces in Vitro. Caries Res..

[64] Kapor S., Rankovic M.J., Khazaei Y., Crispin A., Schüler I., Krause F., Lussi A., Neuhaus K., Eggmann F., Michou S. (2021). Systematic Review and Meta-Analysis of Diagnostic Methods for Occlusal Surface Caries. Clin. Oral Investig..

[65] Thanh M.T.G., Van Toan N., Toan D.T.T., Thang N.P., Dong N.Q., Dung N.T., Hang P.T.T., Anh L.Q., Tra N.T., Ngoc V.T.N. (2021). Diagnostic Value of Fluorescence Methods, Visual Inspection and Photographic Visual Examination in Initial Caries Lesion: A Systematic Review and Meta-Analysis. Dent. J..

[66] Sadatullah S., Mohamed N.H., Razak F.A. (2012). The Antimicrobial Effect of 0.1 Ppm Ozonated Water on 24-Hour Plaque Microorganisms in Situ. Braz. Oral Res..

[67] Epelle E.I., Macfarlane A., Cusack M., Burns A., Thissera B., Mackay W., Rateb M.E., Yaseen M. (2022). Bacterial and Fungal Disinfection via Ozonation in Air. J. Microbiol. Methods.

[68] Gupta S., Deepa D. (2016). Applications of Ozone Therapy in Dentistry. J. Oral Res. Rev..

[69] International Scientific Committee of Ozone Therapy—ISCO3 Learning Methodology Instructions and Perfection in Ozone Therapy for Medical Doctors 2015. https://isco3.org/wp-content/uploads/2015/09/ISCO3-HUM-00-01.pdf.

