# Supplementary material for: Ozone Treatment for the Management of Caries in Primary Dentition: A Systematic Review of Clinical Studies
Source: Dent J (Basel). 2024 Mar 6;12(3):69. doi: 10.3390/dj12030069 (PMC10969751; doi:10.3390/dj12030069)
Supplement: Supplementary file 1 [file dentistry-12-00069-s001.zip › dentistry-2868002-supplementary.pdf]

Supplementary material

Manuscript title: Ozone Treatment for the Management of Caries in Primary Dentition: A Systematic Review of Clinical Studies.

Authors: Federica Veneri, Tommaso Filippini, Ugo Consolo, Marco Vinceti, Luigi Generali.

Supplementary Table S1. Criteria adopted for risk of bias assessment using the current versions of the Cochrane RoB 2 for randomized trials.

Domains	Criteria
D1. Bias due to randomization	Studies are considered at "low risk of bias" if the group allocation was randomized, and randomization process was clearly defined and concealed until allocation. Studies are considered at "some concerns of bias" if group allocation was randomized, the randomization process was clearly defined, but not concealed until allocation. Studies are considered at "high risk of bias" if information about random allocation process were not reported.
D2. Bias due to deviation from intended intervention	Studies are considered at "low risk of bias" if the type of interventions (including protocol, timings, concentration of agents, or other non-protocol interventions, if applicable) are clearly specified. Studies are considered of "some concerns" if information regarding interventions are partially described. Studies are considered at "high risk of bias" if information regarding intervention protocols are not clearly reported or if there is no control group.
D3. Bias due to missing outcome data	Studies are considered at "low risk of bias" if less than 10% of participants were excluded due to missing data, while at "some concerns of bias" of bias if from 10-20%. Studies with higher proportion (≥20%) or not reporting information on this are considered at "high risk of bias".
D4. Bias due to measurement of the outcome	Studies are considered at "low risk of bias" if outcome assessment was based on validated criteria and/or direct measurement of caries-related parameters (e.g. bacterial count, fluorescence). Studies are considered at "some concerns of bias" if outcome assessment was based on clinical examination according to non-validate tools. Studies are considered at "high risk of bias" if outcome measures were not specified.
D5. Bias in selection of reported results	Studies are considered at "low risk of bias" if all outcomes relevant for the evaluation, as outlined in the protocol and/or in the methods, are reported in sufficient detail. Studies are considered at "some concerns of bias" if some outcomes outlined in the protocol and/or in the methods are not reported. Studies are considered at "high risk of bias" if no protocol was available, and a prior plan was not outlined in the methods.
Overall risk of bias	If all domains were at "low risk of bias", the overall risk was considered "low". If at least one domain was found at "some concerns of bias", the overall risk was considered "some concerns of bias" meaning there is some concern about bias in the result, although it is not clear that there is an important risk of bias. If at least one domain was found at "high risk of bias" or 3 or more domains are at "some concerns of bias", the overall risk was considered "high".

Supplementary Table S2. Criteria adopted for risk of bias assessment using the current versions of the Cochrane ROBINS-I tool (Risk Of Bias In Non-randomized Studies - of Interventions).

Domains	Criteria
D1. Bias due to confounding	Studies are considered at "low risk of bias" if they considered age* and oral hygiene status** in the management of confounding factors or if they had split-mouth design. Studies are considered at "some concerns of bias" if they considered one between age and oral hygiene status. Studies are considered at "high risk of bias" if factors adjusted for – if any are not reported.<br>*Age matching among intervention groups is accounted for as "adjustment" if a range of maximum 5 years of age of participants is considered or if similar mean age and standard deviation is reported **oral hygiene status matching among intervention groups is accounted for as "adjustment" if intervention groups have a similar hygiene status, according to validated scales.
D2. Bias in selection of participants in the study	Studies are considered at "low risk of bias" if selection of eligible participants/carious lesions was based on characteristics observed before the start of intervention. Studies are considered at "some concerns" if selection of eligible participants/carious lesions was based on characteristics observed after the start of intervention. Studies are considered at "high risk" if inclusion criteria of participants/carious lesions were not clearly reported.
D3. Bias in classifications of interventions	Studies are considered at "low risk of bias" if the type of interventions (including protocol, timings, concentration of agents, if applicable) are clearly specified. Studies are considered of "some concerns" if information regarding interventions are partially described. Studies are considered at "high risk of bias" if information regarding intervention protocols are not clearly reported or if there is no control group.
D4. Bias due to deviations from the intended interventions	Studies are considered at "low risk of bias" if management of possible non-protocol type of interventions (e.g. abstention from other treatments, recommendation for home hygiene procedures, if applicable) is clearly specified. Studies are considered of "some concerns" if management of non-protocol type of interventions (if applicable) is partially described. Studies are considered at "high risk of bias" if information regarding possible non-protocol interventions are not reported.
D5. Bias due to missing data	Studies are considered at "low risk of bias" if less than 10% of participants were excluded due to missing data, while at "some concerns of bias" if from 10-20%. Studies with higher proportion (≥20%) or not reporting information on this are considered at "high risk of bias".
D6. Bias due to outcome measurement	Studies are considered at "low risk of bias" if outcome assessment was based on validated criteria and/or direct measurement of caries-related parameters (e.g. bacterial count, fluorescence). Studies are considered at "some concerns of bias" if outcome assessment was based on clinical examination according to non-validate tools. Studies are considered at "high risk of bias" if outcome measures were not specified.
D7. Bias in selection of reported results	Studies are considered at "low risk of bias" if all outcomes relevant for the evaluation, as outlined in the protocol and/or in the methods, are reported in sufficient detail. Studies are considered at "some concerns of bias" if some outcomes outlined in the protocol and/or in the methods are not reported. Studies are considered at "high risk of bias" if no protocol was available, and a prior plan was not outlined in the methods.
Overall risk of bias	If all domains were at "low risk of bias", the overall risk was considered "low". If at least one domain was found at "some concerns of bias", the overall risk was considered "some concerns of bias" meaning there is some concern about bias in the result, although it is not clear that there is an important risk of bias. If at least one domain was found at "high risk of bias" or 3 or more domains are at "some concerns of bias", the overall risk was considered "high".
